# 

**Published:** 1888-09-15

**Authors:** 


					jWCE ONE PENNY.
I 1?3, Vol. IV.-
-Sept. 15th, 1888.
^^tered at the General Post Office
as a Newspaper.]
u
Per Annum, 6s. 6d.. post free.
fHfi
Monthly Farts, 6d.; post free, 7id,
Ditto per Ann.. 7s. 6d. post free.
weV J'Ri fife.
'/.ascoiv Western InfirmahiT
Pi
/^IWW A Weekly Institutional Journal of
Sttottf, /Iftcbtchie, IRursmg, ani) fl^Uatttljropa.

				

